# Biofilm Formation and Detection of Fluoroquinolone- and Carbapenem-Resistant Genes in Multidrug-Resistant *Acinetobacter baumannii*

**DOI:** 10.1155/2019/3454907

**Published:** 2019-12-20

**Authors:** María-Guadalupe Avila-Novoa, Oscar-Alberto Solís-Velázquez, Daniel-Eduardo Rangel-López, Jean-Pierre González-Gómez, Pedro-Javier Guerrero-Medina, Melesio Gutiérrez-Lomelí

**Affiliations:** Laboratorio de Alimentos, Departamento de Ciencias Médicas y de la Vida, División de Desarrollo Biotecnológico, Universidad de Guadalajara, Centro Universitario de la Ciénega. Av. Universidad 1115, Col. Linda Vista, 47820 Ocotlán, Jalisco, Mexico

## Abstract

*Acinetobacter baumannii* is an important opportunistic pathogen that shows resistance to cephalosporins, penicillins, carbapenems, fluoroquinolones, and aminoglycosides, the multiresistance being associated with its ability to form biofilms in clinical environments. The aim of this study was to determine biofilm formation and its potential association with genes involved in antibiotic resistance mechanisms of *A. baumannii* isolates of different clinical specimens. We demonstrated 100% of the *A. baumannii* isolates examined to be multidrug resistant (MDR), presenting a 73.3% susceptibility to cefepime and a 53.3% susceptibility to ciprofloxacin. All *A. baumannii* isolates were positive for *bla*_OXA-51_, 33.3% being positive for *bla*_OXA-23_ and IS*Aba1*, and 73.3% being positive for *gyrA*. We found 86.6% of *A. baumannii* strains to be low-grade biofilm formers and 13.3% to be biofilm negative; culturing on Congo red agar (CRA) plates revealed that 73.3% of the *A. baumannii* isolates to be biofilm producers, while 26.6% were not. These properties, combined with the role of *A. baumannii* as a nosocomial pathogen, increase the probability of *A. baumannii* causing nosocomial infections and outbreaks as a complication during therapeutic treatments and emphasize the need to control *A. baumannii* biofilms in hospital environments.

## 1. Introduction

*A. baumannii* is an important opportunistic nosocomial pathogen that causes epidemic pneumonia, urinary tract infections, septicemia, and meningitis. Susceptibility of *A. baumannii* isolates to carbapenems, third- or fourth-generation cephalosporins, fluoroquinolones, and aminoglycosides is less than 30%, which presents a key challenge for empirical therapeutic choice; inappropriate treatments have clearly been associated with increased mortality and healthcare costs [1–3]. The SENTRY Antimicrobial Surveillance Program in 2017 [4] had reported the overall highest frequency of extensively multidrug-resistant (MDR) *A. baumannii* isolates to occur in Europe (66.4%), followed by Latin America (61.5%), Asia-Pacific (60.8%), and North America (38.8%), based on a total of 15,491 *A. baumannii* group (ACBg) isolates collected from over 200 medical locations between 1997 and 2016. *Acinetobacter* sp. is much more prevalent, presenting higher rates of antimicrobial resistance in Latin America than in other regions. An important mechanism of resistance for MDR strains of *A. baumannii* is linked to their ability to produce biofilms [5]. Therefore, *A. baumannii*, especially MDR ones, has gradually gained attention as a human pathogen in hospital environment [6]. Recent studies have reported the biofilm-forming ability of *A. baumannii* strains to be related to major virulence factors, promoting bacterial persistence and chronicity in a specific manner, distinct from the MDR phenotypes. In addition, quorum sensing, which is a communication mechanism used by bacteria to recognize population density fluctuations and control gene expression [7], has been shown to be associated with biofilm formation by *A. baumannni* [8–10]. Among the different *A. baumannii* virulence factors, the most important is their ability to produce biofilms and their survival in hospital environments, which is related to their high degree of antibiotic resistance [11, 12]. Therefore, this study aimed to investigate clinical *A. baumannii* isolates in terms of biofilm formation and their potential association with genes involved in antibiotic resistance mechanisms.

## 2. Materials and Methods

### 2.1. Bacterial Strains and Antibiotic Sensitivity Testing

Different clinical specimens obtained during the month of September 2018, including wounds, urinary catheters, blood, tracheal secretions, bronchoalveolar lavages, and sputum, were inoculated onto blood agar and MacConkey agar plates for 24 h at 33°C. Of the 40 bacterial isolates recovered from these clinical specimens, 15 strains of *A. baumannii* were selected for further studies. The *A. baumannii* strains were identified based on standard bacteriological tests including Gram staining, oxidase and catalase activity, motility, liquefaction of gelatin, lysine decarboxylase, ornithine decarboxylase, citrate utilization, oxidative/fermentative (O/F) glucose tests, and growth ability at 44°C [13]. The findings were confirmed using PCR based on the intergenic spacer region of the 16S–23S rRNA genes [14]. Patterns of resistance and/or susceptibility were determined using the agar diffusion method, according to the American Clinical Laboratory Standardization Committee (CLSI) [15]. The antibiotics used were cefepime (FEP: 30 *μ*g), ciprofloxacin (CIP: 5 *μ*g), amikacin (AMK: 30 *μ*g), piperacilin-tazobactam (PTZ: 100/10 *μ*g), trimethoprim-sulfamethoxazole (SXT: 2.5/23.75 *μ*g), imipenem (IPM: 10 *μ*g), erythromycin (E: 15 *μ*g), dicloxacillin (DC: 1 *μ*g), and cloxacillin (CX: 1 *μ*g) (BD BBL “Sensi-Disc”). The isolates were cultured on Mueller Hinton agar plates (BD Diagnostic Systems) inoculated with a bacterial suspension equal to 0.5 McFarland and incubated at 37°C for 24 h. Diameters of the zone of inhibition were interpreted with reference to the standards set by the CLSI to determine whether the bacterium was susceptible (S), intermediate (I), or resistant (R) to the tested drugs [15]. Isolates resistant to at least three classes of antibiotics were defined as multidrug-resistant *A. baumannii* (MDR-AB) [16]. *A. baumannii* ATCC 19606 was used as the positive control [17].

### 2.2. Detection of Genes (*bla*_OXA23_*, bla*_OXA51,_*gyrA*, and IS*Aba1*)

Genomic DNA extraction from *A. baumannii* was performed using the protocol described by Pu et al. [18]. Detection of the group of OXA-carbapenemases (bla_OXA-23-like_ and bla_OXA-51-like_) was performed as reported by Woodford et al. [19]. The conditions used were as follows: 5 min at 94°C, followed by 30 cycles of 45 s at 94°C, 1 min at 52°C, and 1 min at 72°C, and a final extension of 6 min at 72°C. Amplification of DNA gyrase A subunit (*gyrA*) was performed according to the protocol described by de la Fuente et al. [20]. The conditions used were as follows: 3 min at 96°C, followed by 24 cycles of 15 s at 96°C, 30 s at 50°C, and 90 s at 70°C, and a final extension of 5 min at 70°C. IS*Aba1* promoter was detected according to the protocol described by Segal et al. [21]. The conditions used were as follows: 5 min at 95°C, followed by 35 cycles of 45 s at 94°C, 45 s at 56°C, and 3 min at 72°C, and a final extension of 5 min at 72°C.  Table 1 lists the primers used for the detection of *bla*_OXA23_, *bla*_OXA51_, *gyrA*, and IS*Aba1. A. baumannii* ATCC 19606, *Pseudomonas aeruginosa* ATCC 15442, and *Escherichia coli* ATCC 25922 were used as reference strains for quality control.

### 2.3. Phenotypic Analysis of Biofilms

Phenotypic characterization was carried out by culturing the isolates on Congo red agar (CRA), as described by Arciola et al. [22]. Three replicates were performed for each strain.

### 2.4. Semiquantitative Adherence Assay

Ability of the strains to form biofilms was investigated by culturing them in 96-well flat-bottomed microtiter polystyrene plates as described by Kouidhi et al. [23]. For each strain, three wells of the microtiter plate were filled with 200 *μ*L bacterial suspension in tryptic soy broth (TSB; BD Diagnostic Systems) with 0.25% glucose (w/v) (TSB + 0.25% G). The plates were then incubated at 37°C for 24 h. Wells filled with broth medium (TSB + 0.25% G) were used as negative controls and *A. baumannii* ATCC 19606 was used as the positive control. The content of each well was subsequently aspirated and washed thrice with phosphate-buffered saline (PBS; 7 mM Na_2_HPO_4_, 3 mM NaH_2_PO_4_, and 130 mM NaCl, pH 7.4) to remove the planktonic bacteria. The attached bacteria were fixed with 95% ethanol for 5 min, after which the plates were emptied and left to dry. The plates were stained with 100 *μ*L of 1% (w/v) crystal violet solution (Hycel, Zapopan, Jalisco, Mexico) per well for 5 min. The excess stain was rinsed off with sterile distilled water, and the microtiter plates were air-dried. Optical density of each well was measured at 570 nm (OD_570_), using a Multiskan FC (Thermo Fisher Scientific Inc., Madison, WI, USA). Biofilm formation was interpreted as highly positive (OD_570_ ≥ 1), low-grade positive (0.1 ≤ OD_570_ < 1), or negative (OD_570_ < 0.1).

## 3. Results

**Figure 1:**
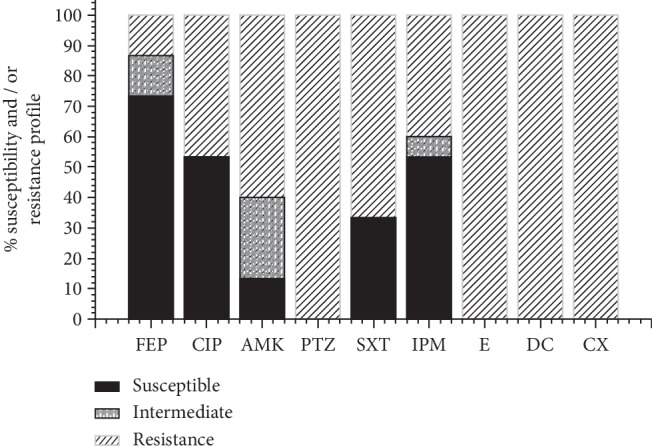
Antimicrobial resistance pattern of *Acinetobacter baumannii* to different antibiotics. FEP, cefepime; CIP, ciprofloxacin; AMK, amikacin; PTZ, piperacillin-tazobactam; STX, trimethoprim-sulfamethoxazole; IMP, imipenem; E erythromycin; DC, dicloxacilin; CX, cloxacilin.

Of the 40 strains, 15 strains of *A. baumannii* were detected from the clinical samples. Isolates of *A. baumannii* presented a pattern of 100% resistance (15/15) to dicloxacillin, cloxacillin, piperacillin/tazobactam, and erythromycin, followed by 66.6% to sulfamethoxazole/trimethoprim (10/15), 60% to amikacin (9/15), 46.6% to ciprofloxacin (7/15), and 40% to imipenem (6/15). The susceptibility test in this study confirmed *A*. *baumannii* isolates to be 73.3% susceptible to cefepime (11/15), 53.3% to imipenem and ciprofloxacin (8/15), and 33.3% to trimethoprim-sulfamethoxazole (5/15) (Figure 1).

**Figure 2:**
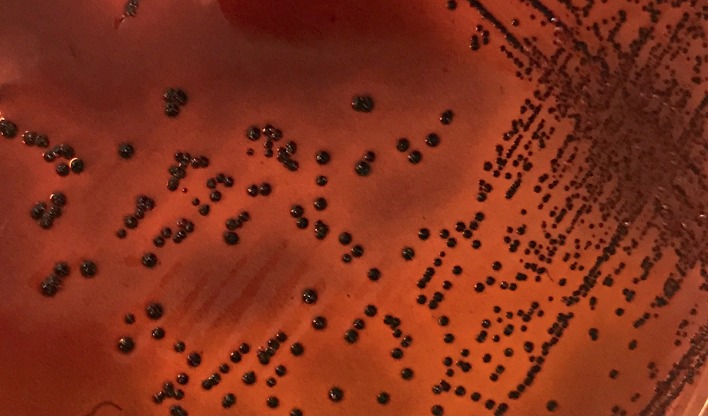
CRA plate test: black colonies of the slime-producing *Acinetobacter baumannii*.

**Figure 3:**
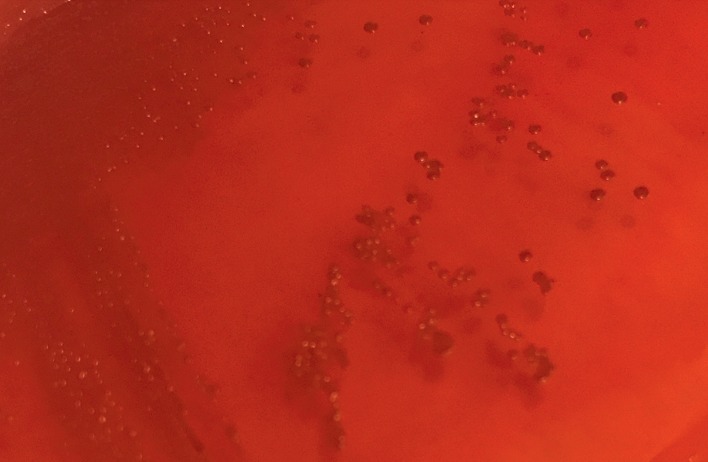
CRA plate test: red colonies of the non-slime-producing *Acinetobacter baumannii*.

Overall, 100% (15/15) of the *A. baumannii* isolates examined were MDR; four of them (*A. baumanni*-7, 8, 11, and 12) were resistant to eight and nine antibiotics, and two of them were susceptible to cefepime (Table 2). In the isolates of *A. baumannii*-3 and *A. baumannii*-8, four genes associated with resistance to imipenem and ciprofloxacin were detected. The genes detected by PCR were *bla*_OXA-51_, *bla*_OXA-23_, IS*Aba1*, and *gyrA* in 100% (15/15), 33.3% (5/15), 33.3% (5/15), and 73.3% (11/15) of the *A. baumannii* isolates, respectively (Table 2). In 46.6% (7/15) of isolates resistant to ciprofloxacin, *gyrA* was detected (Figure 1, Table 2). Finally, the OD_570_ results showed 86.6% (13/15) of the *A. baumannii* strains to be low-grade biofilm formers (0.1 ≤ OD_570_ < 1) and 13.3% (2/15) to be biofilm negative (OD_570_ < 0.1). On CRA, 73.3% (11/15) of the *A. baumannii* isolates were biofilm producers (Figure 2), while 26.6% (4/15) were not (Figure 3).

## 4. Discussion

*A. baumannii* is a nosocomial pathogen causing multiple pathologies, where biofilm plays a role in the colonization during infection, thus providing an opportunity for *A. baumannii* to develop drug resistance. In this study, 100% of *A. baumannii* isolates were resistant to a wide range of antibiotic groups, including third-generation cephalosporins, fluoroquinolones, aminoglycosides, and carbapenems (Figure 1). Carbapenems are a subgroup of beta-lactams, among which imipenem treatment was confirmed to be effective. However, many current studies have reported increasing resistance to imipenem and ciprofloxacin. Di-Domenico et al. [10] found *A. baumannii* isolates from patients with colonized skin ulcers to have 75% resistance to imipenem, 75% resistance to trimethoprim-sulfamethoxazole, and 91% resistance to ciprofloxacin. Addi-Ali et al. [12] showed 92% of *A. baumannii* clinical isolates to be resistant to ciprofloxacin and 68% to be resistant to imipenem. Results of the current study (*bla*_OXA-51_ (100%), *bla*_OXA-23_ (33.3%), IS*Aba1* (33.33%), and *gyrA* (73.3%)) are consistent with those of other studies, which had reported the most prevalent carbapenem hydrolyzing *β*-lactamases genes in *A. baumannii* to include *bla*_OXA-51_ (83–100%) and *bla*_OXA-23_ (59–96%) [16, 24, 25]. Therefore, the most prevalent mechanism underlying the resistance of *A. baumannii* to carbapenem antibiotics is the production of OXA-type *β*-lactamases, and their resistance to quinolones is related to alterations in the target enzymes GyrA and ParC [13, 26]. However, the rapid emergence of resistance to aminoglycosides in clinical isolates of *Acinetobacter* has been linked to their ability to acquire resistance through transposons, plasmids, and integrons; several factors, such as geographical region, misuse of antibiotics, and inappropriate prescription of aminoglycosides, can play a significant role in the prevalence of aminoglycoside resistance genes [16, 27]. One of the most important mechanisms in the development of an MDR strain is the bacterial biofilm formation, which has attracted extensive study in recent years, since *A. baumannii* clinical isolates possess a strong ability to form biofilms, which in turn is associated with a significant increase in the antibiotic resistance of the bacteria [17]. Antimicrobial susceptibility testing showed 100% of the *A. baumannii* strains to be MDR, having the ability to form biofilms. Thus, the ability of MDR *A. baumannii* strains to form biofilms, which limit the diffusion of antibiotics to the site of action due to its components or alter the phenotypes or genotypes of the strains, favors resistance. Babapour et al. [28] had shown 92% of the biofilm-forming *A. baumannii* clinical isolates from patients with nosocomial infections in three hospitals in Tehran to be MDR, and 86% to be extensively drug-resistant. Biofilm formation, a factor contributing to the virulence of *A. baumannii,* is associated with long-term persistence in hospital environments [29]. Yang et al. [30] had argued that the factors leading to enhanced antibiotic resistance in the biofilm phenotype include impaired drug diffusion due to microbial aggregations, overexpression of the exopolymeric substance (EPS) matrix, alterations in microbial phenotypic and genotypic features due to stress responses, and physiological heterogeneity due to physicochemical gradients and persisters. However, biofilm formation depends on an interaction between three main components: the bacterial cells, the attachment surface, and the surrounding medium [31].

A limitation of this study was the lack of serotyping data for the isolates of *A. baumannii* or components of the extracellular matrix (exopolysaccharides, eDNA, proteins, and lipids) of multicellular communities, such as the biofilms formed by nosocomial pathogens. Biofilm composition provides a basis for the development of better strategies to reduce sources of contamination in the hospital setting.

Overall, this could lead to the incorporation of new therapeutic strategies that take into account the behavior of sessile cells and the mechanisms of antimicrobial resistance in *A. baumannii* strains of different origin, with the aim to incorporate or modify the therapeutic treatment schemes used in the control of this nosocomial pathogen or implement cleaning and disinfection procedures to improve hospital environments.

## 5. Conclusions

In the majority of the isolates of multidrug-resistant *A. baumannii, bla*_OXA-51_ + *bla*_OXA-23_ were detected as the determinant factor for carbapenemic resistance having a direct relation with biofilm formation. These studies provide useful information for new therapeutic regimes in *A. baumannii* infections.

## Figures and Tables

**Table 1:** Sequences of primers used for PCR in this study.

Genes	Primers	Sequences (5′-3′)	Product sizes (base pairs)
*gyrA*	GyrA-F	5′-AAATCTGCCCGTGTCGTTGGT-3′	343
	GyrA-R	5′-GCCATACCTACGGCGATACC-3′	

*blaOXA-23*	OXA-23-like F	5′-GATCGGATTGGAGAACCAGA-3′	501
	OXA-23-like R	5′-ATTTCTGACCGCATTTCCAT-3′	

*blaOXA-51*	OXA-51-like F	5′-TAATGCTTTGATCGGCCTTG-3′	353
	OXA-51-like R	5′-TGGATTGCACTTCATCTTGG-3′	

*ISAba1*	ISAba 1F	5′-CACGAATGCAGAAGTTG-3′	549
	ISAba 1R	5′-CGACGAATACTATGACAC-3′	

**Table 2:** Characteristics of the multidrug resistance of *Acinetobacter baumannii* to different antibiotics.

Strains	Multidrug resistance	Genes
*A. baumannii*-1	CIP-AMK-DC-CX-E	*bla*_*OXA-51*_ + *bla*_*OXA-23*_ + *gyrA*
*A. baumannii*-2	AMK-DC-CX-SXT-E-IPM	*bla*_*OXA-51*_ + ISA*ba1*
*A. baumannii*-3	CIP-DC-CX-PTZ-SXT-E-IPM	*bla*_*OXA-51*_ + *bla*_*OXA-23*_ + ISA*ba1* + *gyrA*
*A. baumannii*-4	DC-CX-E	*bla*_*OXA-51*_ + *gyrA*
*A. baumannii*-5	DC-CX-PTZ-E	*bla*_*OXA-51*_ + *gyrA*
*A. baumannii*-6	DC-CX-PTZ-SXT-E	*bla*_*OXA-51*_
*A. baumannii*-7	CIP-AMK-DC-CX-PTZ-SXT-E-IPM	*bla*_*OXA-51*_ + *gyrA*
*A. baumannii*-8	FEP-CIP-AMK-DC-CX-PTZ-SXT-E-IPM	*bla*_*OXA-51*_ + *bla*_*OXA-23*_ + ISA*ba1* + *gyrA*
*A. baumannii*-9	FEP-DC-CX-PTZ-SXT-E-IPM	*bla*_*OXA-51*_ + *bla*_*OXA-23*_ + ISA*ba1*
*A. baumannii*-10	CIP-DC-CX-PTZ-E	*bla*_*OXA-51*_ + *gyrA*
*A. baumannii*-11	CIP-AMK-DC-CX-PTZ-SXT-E-IPM	*bla*_*OXA-51*_ + *gyrA*
*A. baumannii*-12	CIP-AMK-DC-CX-PTZ-SXT-E-IPM	*bla*_*OXA-51*_ + ISA*ba1* + *gyrA*
*A. baumannii*-13	AMK-DC-CX-PTZ-SXT-E	*bla*_*OXA-51*_ + *gyrA*
*A. baumannii*-14	AMK-DC-CX-PTZ-SXT-E	*bla*_*OXA-51*_ + *gyrA*
*A. baumannii*-15	AMK-DC-CX-PTZ-SXT-E	*bla*_*OXA-51*_ + *bla*_*OXA-23*_

FEP, cefepime; CIP, ciprofloxacin; AMK, amikacin; PTZ, piperacillin-tazobactam; STX, trimethoprim-sulfamethoxazole; IMP, imipenem; E, erythromycin; DC, dicloxacillin; CX, cloxacillin.

## Data Availability

The data used to support the findings of this study are included within the article.
